# Usefulness of monocyte distribution width and presepsin for early assessment of disease severity in COVID-19 patients

**DOI:** 10.1097/MD.0000000000029592

**Published:** 2022-07-08

**Authors:** Sei Won Kim, Heayon Lee, Sang Haak Lee, Sung Jin Jo, Jehoon Lee, Jihyang Lim

**Affiliations:** a Division of Pulmonary, Critical Care and Sleep Medicine, Department of Internal Medicine, Eunpyeong St. Mary’s Hospital, College of Medicine, The Catholic University of Korea, Seoul, Republic of Korea; b Department of Laboratory Medicine, Eunpyeong St. Mary’s Hospital, College of Medicine, The Catholic University of Korea, Seoul, Republic of Korea.

**Keywords:** monocyte distribution width, MDW, presepsin, KL-6, SARS-CoV-2

## Abstract

Early predictors of severe coronavirus disease 2019 (COVID-19) would identify patients requiring intensive care. Recently, the monocyte distribution width (MDW) and presepsin level have been used for the early diagnosis of sepsis. Here, we assessed the utility of MDW and presepsin for the early assessment of COVID-19 severity.

Eighty-seven inpatients with confirmed COVID-19 were enrolled and divided into 3 groups by the type of respiratory support: (1) mechanical ventilation or high-flow nasal cannula oxygen therapy (MVHF-OT), (2) conventional oxygen therapy, and (3) no oxygen therapy. We measured the complete blood count; MDW; erythrocyte sedimentation rate; and the levels of presepsin, C-reactive protein, procalcitonin, lactate dehydrogenase, ferritin, Krebs von den Lungen-6 (KL-6), and severe acute respiratory syndrome coronavirus 2 (SARS-CoV-2) neutralizing antibody.

Thirteen (14.9%) patients on MVHF-OT exhibited a significantly higher mortality and a longer hospital stay than did the others. The MDW and presepsin levels were significantly elevated on admission, and correlated with COVID-19 severity (both *P* < .001). Notably, only the MDW correlated significantly with symptoms in the no oxygen therapy group (*P* < .012). In the first week after admission, the MDW fell and no longer differed among the groups. The KL-6 level did not differ by disease severity at any time. Neutralizing antibodies were detected in 74 patients (91.4%) and the level of neutralization correlated significantly with COVID-19 severity (*P* < .001).

The MDW and presepsin are useful indicators for early assessment of disease severity in COVID-19 patients.

## 1. Introduction

In the time since the first coronavirus disease 2019 (COVID-19) cases in Wuhan, China, 151,803,822 cases and 3,186,538 deaths have been reported worldwide as of May 3, 2021.^[1]^ Most patients have mild or nonspecific symptoms; some are asymptomatic.^[2]^ Severe symptoms develop in approximately 14% of patients; the mortality rate is about 2%,^[3]^ thus much lower than that associated with Middle East respiratory syndrome coronavirus (MERS-CoV) (about 35%).^[4]^ However, MERS-CoV cases and deaths numbered only 2468 and 851 from April 2012 to September 2019.^[5]^ As the COVID-19 incidence continues to rise, the medical burden is increasing. In March and April 2020, in New York City, 14.2% of 2634 patients were treated in intensive care units, 12.2% received invasive mechanical ventilation, and 21% died.^[6]^ Obesity, hypertension, diabetes, and other comorbidities were associated with severe or fatal outcomes.^[7]^ Several laboratory biomarkers, including lymphopenia and the levels of C-reactive protein (CRP), procalcitonin (PCT), and lactate dehydrogenase (LDH), have been used to stratify disease severity.^[8,9]^ An early biomarker of severity would identify patients requiring hospitalization or intensive care. Recently, the monocyte distribution width (MDW) and presepsin level were suggested to be useful early biomarkers of sepsis.^[10–12]^ MDW reflects the heterogeneity in the size of circulating monocytes, which play important roles in the pathogenesis during the early stage of infection and sepsis.^[13]^ After infectious stimuli, monocytes undergo activation leading to functional and morphological changes.^[14]^ Presepsin, a 13-kDa protein and a fragment of monocyte LPS receptor CD14, is released in the blood circulation by proinflammatory signals during infection and has diagnostic and prognostic values in sepsis.^[15,16]^ Here, we explored the usefulness of MDW and presepsin for early assessment of disease severity in COVID-19 patients.

## 2. Methods

### 2.1. Patients

We retrospectively analyzed 87 inpatients with confirmed COVID-19 admitted to Eunpyeong St. Mary’s Hospital, College of Medicine, The Catholic University of Korea.

All were diagnosed with severe acute respiratory syndrome coronavirus 2 (SARS-CoV-2) infections via real-time reverse transcription-polymerase chain reaction (rRT-PCR). We included COVID-19-confirmed patients ≥18 years of age for whom biochemical and clinical data were available. Our institutional review board approved the study (approval no. PC21RASE0026).

### 2.2. Clinical characteristics

We recorded patient sex, age, Charlson Comorbidity Index (CCI) score, fever status, respiratory symptoms, pneumonia (or not) at admission, and days from symptom onset to admission. After discharge, mortality and the length of hospital stay were calculated. Patients were divided into 3 groups by COVID-19 severity: (1) mechanical ventilation or high-flow nasal cannula oxygen therapy (MVHF-OT), (2) conventional oxygen therapy (C-OT), and (3) no oxygen therapy (N-OT). The symptoms of the latter group were recorded.

### 2.3. SARS-CoV-2 rRT-PCR

Nasopharyngeal and oropharyngeal swabs were collected into T-SWAB TRANSPORT UTMs (Noble Biosciences, Korea) and sputum specimens into 50-mL Falcon tubes containing phosphate buffer (Corning Inc., USA). A QIAamp DSP Viral RNA Minikit (Qiagen GmbH, Germany), QIAcube System (Qiagen GmbH), NX-48 Viral NA Kit (Genolution, Korea), and Nextractor NX-48 System (Genolution) were used for RNA extraction according to the manufacturers’ instructions. SARS-CoV-2 nucleic acid was amplified by rRT-PCR using PowerCheck 2019-nCoV Real-time PCR Kits (Kogenebiotech, Korea). The ABI 7500 real-time PCR system (Applied Biosystems, USA) was used to amplify the *E* and *RdRp* genes of SARS-CoV-2 (40 cycles). SARS-CoV-2 infection was diagnosed when both genes were detected under 35.0 cycles.

### 2.4. Laboratory findings

We recorded the following at admission and in the first week after admission: white blood cell (WBC), neutrophil, lymphocyte, and monocyte counts; neutrophil to lymphocyte ratio (NLR); hemoglobin level; platelet count; platelet to lymphocyte ratio (PLR); and MDW. All were obtained with the aid of a UniCel DxH 900 Analyzer (Beckman Coulter, USA). We also recorded the erythrocyte sedimentation rate (ESR) and CRP level. The PCT, LDH, and ferritin levels were measured only at admission. Presepsin levels were measured on admission using a PATHFAST Presepsin Kit (Mitsubishi Chemical, Japan). Krebs von den Lungen-6 (KL-6) serum levels were measured using KL-6 ELISA Kits (Mybiosource, USA) at admission and discharge. The levels of circulating neutralizing antibodies against SARS-CoV-2 were determined on discharge using SARS-CoV-2 Surrogate Virus Neutralization Test Kits (GenScript, USA). The SARS-CoV-2-neutralizing antibody test was considered positive if the extent of inhibition (neutralization) was > 20%.

### 2.5. Statistical analyses

Normally distributed continuous variables are presented as means with standard deviations and non-normally distributed continuous variables as medians with interquartile ranges (IQRs: 25th–75th). Categorical data are described as numbers with percentages (%). To compare clinical characteristics and laboratory findings, normally distributed data were subjected to a 1-way analysis of variance with the Tukey post hoc test. The Kruskal-Wallis test and Dunn post hoc test were employed to compare non-normally distributed data. Categorical variables were compared using the chi-squared or Fisher exact test, as appropriate. Missing values were excluded from analysis. All analyses were performed using R ver. 3.1.1 software. The *P* value < .05 was considered statistically significant.

## 3. Results

### 3.1. Basic characteristics of the inpatients

Of the 87 inpatients, 50.6% were male and the mean age was 56.5 ± 17.5 years (Table 1). The median CCI was 2.0 (IQR: 0.0–3.0). Age and the CCI score differed significantly among the groups (*P* = .035 and *P* = .006, respectively). On post hoc analysis, the N-OT group was significantly younger and had a lower CCI score than the C-OT group. Also, the N-OT group exhibited a lower pneumonia rate than the other 2 groups (*P* < .001). The median time between symptom onset and hospital admission was 5.0 days (3.0–9.0) for all groups. Both survival and duration of hospitalization differed significantly by disease severity (*P* = .002 and *P* < .001, respectively).

**Table 1:** Basic characteristics of the inpatients (N = 87).

	MVHF-OT (n = 13)	C-OT (n = 18)	N-OT (n = 56)	Total (N = 87)	*P*
Male (n, %)	9 (69.2%)	10 (55.6%)	25 (44.6%)	44 (50.6%)	.249
Age (yrs)	59.9 ± 15.5	65.7 ± 15.8	52.8 ± 17.4	56.5 ± 17.5	**.035**
CCI (score)	3.0 [1.0; 4.0]	3.0 [1.0; 5.0]	1.0 [0.0; 2.0]	2.0 [0.0; 3.0]	**.006**
Fever* (n, %)	10 (76.9)	13 (72.2)	29 (51.8)	52 (59.8)	.120
Respiratory symptoms* (n, %)	13 (100.0)	16 (88.9)	39 (69.6)	68 (78.2)	**.027**
Pneumonia* (n, %)	13 (100.0)	18 (100.0)	24 (42.9)	55 (63.2)	**<.001**
Interval between symptom onset and admission† (d)	5.0 [4.0; 8.0]	5.0 [2.5; 7.5]	6.0 [3.0; 9.0]	5.0 [3.0; 9.0]	.622
Survival‡ (n, %)	10 (76.9)	17 (94.4)	56 (100.0)	83 (95.4)	**.002**
Hospitalization period (d)	22.0 [17.0;32.0]	15.0 [13.0;20.0]	10.0 [8.0;14.5]	13.0 [9.0;17.5]	**<.001**

The statistically significant values were shown in bold.

CCI = Charlson Comorbidity Index, C-OT = conventional oxygen therapy, COVID-19 = coronavirus disease 2019, MVHF-OT = mechanical ventilation or high-flow nasal cannula oxygen therapy, N-OT = no oxygen therapy.

* At admission.

† Patients with no COVID-19 related symptoms or no medical record prior to admission were excluded.

‡ Survival at hospital discharge.

### 3.2. Laboratory findings at admission

**Figure 1.**
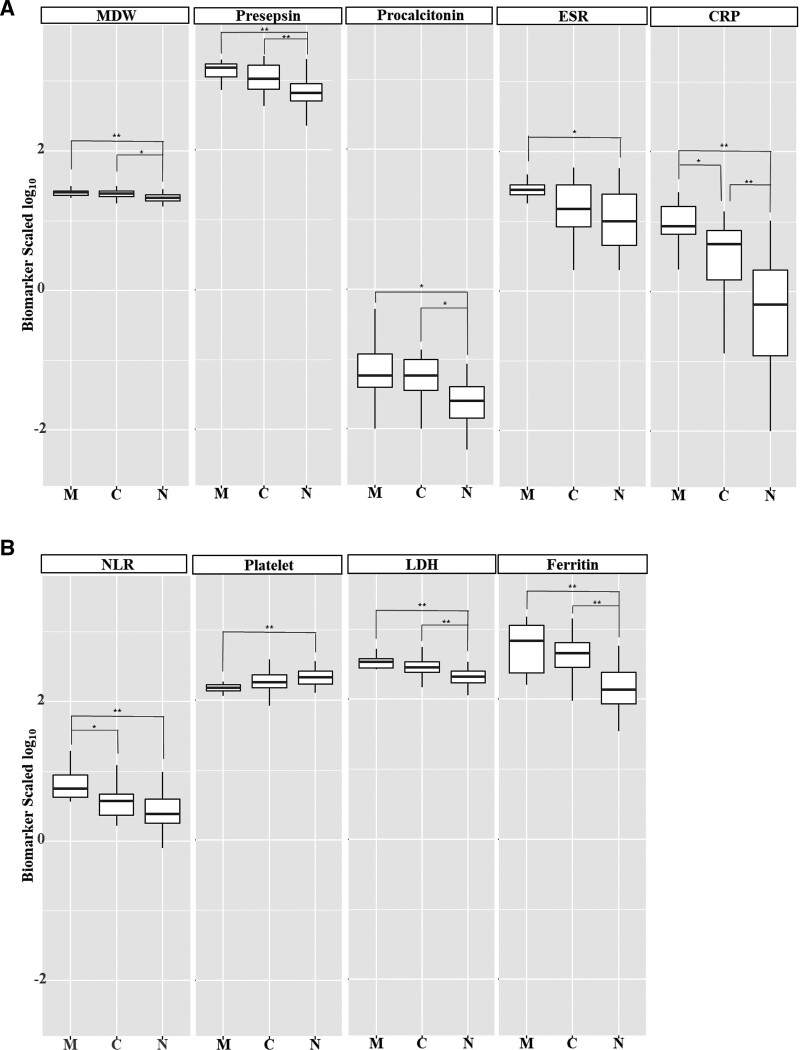
Laboratory data obtained at admission for 3 groups of COVID-19 patients (n = 87). (A) Representative inflammatory markers. (B) Other laboratory findings. M, MVHF-OT; C, C-OT; N, N-OT. *P < .05, **P < .005. C-OT = conventional oxygen therapy, CRP = C-reactive protein, ESR = erythrocyte sedimentation rate, LDH, lactate dehydrogenase, MVHF-OT = mechanical ventilation or high-flow nasal cannula oxygen therapy, MDW = monocyte distribution width, NLR = neutrophil/lymphocyte ratio, N-OT = no oxygen therapy.

The WBC and neutrophil counts did not differ among the groups (Table 2). The MDW and NLR differed by disease severity (both *P* < .001). The MDWs were 25.79 ± 3.92 in the MVHF-OT group, 24.19 ± 3.43 in the C-OT group, and 21.61 ± 3.09 in the N-OT group. The lymphocyte and platelet counts fell with disease severity (*P* < .001 and *P* = .001, respectively). The presepsin, CRP, and PCT levels, and the ESR, differed by disease severity (*P* < .001, *P* = .015, *P* < .001, and *P* < .001, respectively). The presepsin levels were 1488 (1096–1702) in the MVHF-OT group, 1051 (710–1656) in the C-OT group, and 654 (501–890 pg/mL) in the N-OT group. The LDH and ferritin levels differed significantly among the groups (both *P* < .001). The data were subjected to post hoc analysis. The levels of MDW, prespesin, and PCT, differed significantly between the N-OT group and the other groups but not between the MVHF-OT and C-OT groups (Fig. 1A). The CRP level differed significantly among the groups (MVHF-OT group 8.68 [6.62–16.60]; C-OT group 4.82 [1.20–7.53]; N-OT group 0.65 [0.12–2.02 mg/dL]). The NLR differed significantly between the MVHF-OT group and the other groups but not between the C-OT and N-OT groups (Fig 1B). The levels of LDH, and ferritin, differed significantly between the N-OT group and the other groups but not between the MVHF-OT and C-OT groups.

**Table 2:** Laboratory findings at admission (N = 87).

	MVHF-OT (n = 13)	C-OT (n = 18)	N-OT (n = 56)	Total (N = 87)	*P*
WBC (×10^9^/L)	7.40 [3.80;9.20]	5.45 [4.70;7.70]	5.30 [4.50;6.40]	5.40 [4.50;7.25]	.576
Neutrophil (×10^9^/L)	6.52 [2.56;7.45]	3.72 [2.76;5.06]	3.35 [2.56;4.47]	3.59 [2.61;4.80]	.134
Lymphocyte (×10^9^/L)	0.72 [0.55;0.85]	0.91 [0.81;1.19]	1.34 [0.90;1.72]	1.12 [0.78;1.52]	**<.001**
Monocyte (×10^9^/L)	0.36 [0.32;0.51]	0.42 [0.35;0.58]	0.39 [0.31;0.52]	0.39 [0.32;0.52]	.518
MDW	25.79 ± 3.92	24.19 ± 3.43	21.61 ± 3.09	22.81 ± 3.65	**<.001**
NLR	5.66 [4.23;8.78]	3.75 [2.22;4.72]	2.46 [1.78;4.09]	3.28 [2.02;4.79]	**<.001**
Hemoglobin (g/dL)	13.73 ± 1.83	13.18 ± 2.06	13.67 ± 1.59	13.58 ± 1.72	.784
Platelet (×10^9^/L)	148.0 [135.0;166.0]	179.5 [149.0;235.0]	213.0 [167.5;261.0]	190.0 [158.5;252.5]	**.001**
PLR	229.35 [169.96;289.86]	178.11 [145.08;206.17]	162.65 [124.98;242.06]	175.05 [129.77;242.97]	.250
Presepsin (pg/mL)	1488.0 [1096.0;1702.0]	1051.0 [710.0;1656.0]	654.0 [501.0;890.0]	830.5 [543.0;1182.5]	**<.001**
ESR (mm/h)	28.0 [22.0;37.5]	15.0 [8.0;33.0]	10.0 [4.5;24.5]	14.0 [7.0;28.0]	**.015**
CRP (mg/dL)	8.68 [6.62;16.60]	4.82 [1.20;7.53]	0.65 [0.12;2.02]	1.67 [0.24;6.07]	**<.001**
Procalcitonin (ng/mL)	0.06 [0.04;0.12]	0.06 [0.04;0.10]	0.03 [0.01;0.04]	0.04 [0.02;0.06]	**<.001**
LDH (U/L)	338.0 [279.0;376.0]	284.5 [239.0;351.0]	208.0 [171.5;254.0]	243.0 [187.0;288.0]	**<.001**
Ferritin(ng/mL)	694.80 [207.30;1227.90]	449.75 [276.40;649.30]	135.60 [85.65;239.30]	178.50 [103.30;359.35]	**<.001**
KL-6 (ng/mL)	0.19 [0.15;0.26]	0.17 [0.15;0.24]	0.16 [0.11;0.23]	0.17 [0.12;0.24]	.292

The statistically significant values were shown in bold.

C-OT = conventional oxygen therapy, CRP = C-reactive protein, ESR = erythrocyte sedimentation rate, KL-6 = Krebs von den Lungen-6., LDH = lactate dehydrogenase, MDW = monocyte distribution width, MVHF-OT = mechanical ventilation or high-flow nasal cannula oxygen therapy, NLR = neutrophil/lymphocyte ratio, N-OT = no oxygen therapy, PLR = platelets/lymphocyte ratio, WBC = white blood cell.

### 3.3. Laboratory findings of the N-OT group on admission by symptoms developing during hospitalization

In the N-OT group (n = 56), the MDW and PCT level were significantly higher in symptomatic than asymptomatic patients (*P* = .012 and *P* = .001, respectively; Table 3). The MDWs were 21.95 ± 3.29 and 20.15 ± 1.42 in the 2 groups, respectively. The ESR and the presepsin, CRP, LDH, and ferritin levels did not differ between the 2 groups.

**Table 3:** Laboratory findings of the N-OT group on admission by symptoms developing during hospitalization (N = 56).

	Symptomatic* (n = 45)	Asymptomatic* (n = 11)	*P*
WBC (×10^9^/L)	5.47 ± 1.66	5.81 ± 1.36	.538
Neutrophil (×10^9^/L)	3.32 [2.51;4.40]	4.34 [2.87;4.68]	.337
Lymphocyte (×10^9^/L)	1.32 [0.87;1.70]	1.40 [1.09;1.64]	.509
Monocyte (×10^9^/L)	0.40 [0.32;0.53]	0.38 [0.23;0.44]	.252
MDW	21.95 ± 3.29	20.15 ± 1.42	**.012**
NLR	2.45 [1.73;3.76]	2.51 [1.89;4.70]	1.000
Hemoglobin (g/dL)	13.80 ± 1.67	13.15 ± 1.12	.223
Platelet (×10^9^/L)	205.0 [167.0;259.0]	252.0 [217.5;267.0]	.117
PLR	161.90 [126.98;249.57]	163.39 [127.53;207.08]	.919
Presepsin (pg/mL)	676.0 [501.0;906.0]	545.0 [494.5;875.5]	.645
ESR (mm/h)	9.0 [4.0;26.0]	19.5 [10.0;24.0]	.450
CRP (mg/dL)	0.68 [0.12;2.16]	0.21 [0.12;1.58]	.371
Procalcitonin (ng/mL)	0.03 [0.02;0.05]	0.01 [0.01;0.02]	**.001**
LDH (U/L)	214.20 ± 55.58	202.18 ± 45.37	.510
Ferritin(ng/mL)	156.1 [84.8;265.4]	104.6 [92.2;130.4]	.204
KL-6 (ng/mL)	0.16 [0.11;0.22]	0.15 [0.10;0.23]	.929

The statistically significant values were shown in bold.

COVID-19 = coronavirus disease 2019, CRP = C-reactive protein, ESR = erythrocyte sedimentation rate, KL-6 = Krebs von den Lungen-6, LDH = lactate dehydrogenase, MDW = monocyte distribution width, NLR = neutrophil/lymphocyte ratio, N-OT = no oxygen therapy, PLR = platelets/lymphocyte ratio, WBC = white blood cell.

* Presence of symptoms related with COVID-19 throughout the entire hospitalization period.

### 3.4. Laboratory findings in the first week after admission

In the first week after admission, the WBC, neutrophil, and lymphocyte counts; the NLR and PLR; and the hemoglobin level differed significantly by disease severity (*P* = .003, *P* < .001, *P* < .001, *P* < .001, *P* = .031, and *P* < .001, respectively; Table 4). However, the MDW, platelet count, and ESR (which differed significantly at admission) did not differ. The CRP level remained significantly different among the groups (*P* < .001). The CRP levels were 5.90 (0.99–11.31) in the MVHF-OT group, 1.78 (0.47–6.85) in the C-OT group, and 0.36 (0.08–1.20 mg/dL) in the N-OT group.

**Table 4:** Laboratory findings in the first week after admission (N = 87).

	MVHF-OT (n = 13)	C-OT (n = 18)	N-OT (n = 56)	Total (N = 87)	*P*
WBC (×10^9^/L)	7.40 [5.80;9.40]	6.25 [4.30;7.50]	5.10 [4.65;6.15]	5.60 [4.80;7.40]	**.003**
Neutrophil (×10^9^/L)	6.44 [4.58;8.44]	3.93 [3.21;5.14]	2.81 [2.23;3.86]	3.45 [2.40;4.79]	**<.001**
Lymphocyte (×10^9^/L)	0.72 [0.61;0.84]	1.24 [0.97;1.58]	1.77 [1.41;2.07]	1.51 [0.96;1.90]	**<.001**
Monocyte (×10^9^/L)	0.48 [0.35;0.61]	0.49 [0.35;0.68]	0.46 [0.40;0.60]	0.46 [0.38;0.61]	.931
MDW	22.43 [19.41;24.23]	21.70 [20.42;23.94]	20.26 [18.90;23.11]	21.09 [19.44;23.71]	.354
NLR	9.37 [5.86;12.91]	3.39 [2.34;5.71]	1.62 [1.19;2.27]	2.21 [1.54;4.40]	**<.001**
Hemoglobin (g/dL)	12.25 ± 1.69	12.36 ± 2.04	13.19 ± 1.51	12.86 ± 1.70	**.031**
Platelet (×10^9^/L)	234.00 ± 88.86	259.50 ± 121.64	264.90 ± 86.54	258.89 ± 94.88	.329
PLR	319.78 [263.98;408.76]	201.55 [142.89;239.34]	141.09 [118.55;189.13]	176.95 [126.04;241.75]	**<.001**
ESR (mm/h)	23.5 [11.0;39.0]	25.0 [11.0;31.0]	18.0 [6.5;33.5]	18.5 [7.0;35.0]	.476
CRP (mg/dL)	5.90 [0.99;11.31]	1.78 [0.47;6.85]	0.36 [0.08;1.20]	0.68 [0.21;2.52]	**<.001**

C-OT = conventional oxygen therapy, CRP = C-reactive protein, ESR = erythrocyte sedimentation rate, KL-6 = Krebs von den Lungen-6, LDH = lactate dehydrogenase, MDW = monocyte distribution width, MVHF-OT = mechanical ventilation or high-flow nasal cannula oxygen therapy, NLR = neutrophil/lymphocyte ratio, N-OT = no oxygen therapy, PLR = platelets/lymphocyte ratio, WBC = white blood cell.

The statistically significant values were shown in bold.

### 3.5. Laboratory findings at discharge

At discharge, neutralizing antibodies were detected in 74 patients (91.4%; Table 5). The KL-6 level did not differ by disease severity, nor did the proportions of patients with SARS-CoV-2-neutralizing antibodies. However, the neutralization rates differed significantly (*P* < .001), being 93.60 (88.80%–98.14%) in the MVHF-OT group, 89.95 (76.50%–96.12%) in the C-OT group, and 66.28 (42.20%–85.84%) in the N-OT group.

**Table 5:** Laboratory findings at discharge (N = 87).

	MVHF-OT (n = 13)	C-OT (n = 18)	N-OT (n = 56)	Total (N = 87)	*P*
KL-6 (ng/mL)	0.19 [0.17;0.20]	0.17 [0.16;0.22]	0.21 [0.16;0.34]	0.19 [0.16;0.30]	.321
Positivity of SARS-CoV-2-neutralizing antibody (n, %)	13 (100.0%)	18 (100.0%)	43 (86.0%)	74 (91.4%)	.093
SARS-CoV-2 neutralization rate (%)	93.60 [88.80;98.14]	89.95 [76.50;96.12]	66.28 [42.20;85.84]	81.10 [51.92;93.60]	**<.001**

C-OT = conventional oxygen therapy = N-OT = no oxygen therapy, KL-6 = Krebs von den Lungen-6, MVHF-OT = mechanical ventilation or high-flow nasal cannula oxygen therapy, SARS-CoV-2 = severe acute respiratory syndrome coronavirus 2.

The statistically significant values were shown in bold.

## 4. Discussion

Early detection of COVID-19 patients who will experience a severe clinical course is important in terms of rapid intensive care and medical attention. We sought early laboratory indicators of COVID-19 severity. The MDW and presepsin level on admission differed significantly by later disease severity. Although the difference was small, the MDW was significantly higher in symptomatic patients not on oxygen therapy than in asymptomatic patients not on such therapy. Certain traditional biomarkers (CRP, PCT, LDH, and ferritin levels) were also useful.

Presepsin is a soluble CD14 protein that modulates the immune response by interacting with T and B cells.^[15]^ The presepsin level is used for early diagnosis and prognostic assessment of patients with systemic infections.^[15]^ Especially in sepsis patients, the serum levels of presepsin are elevated before those of procalcitonin or IL-6; presepsin serves as a biomarker of sepsis.^[16,17]^ Carpio et al^[18]^ set presepsin thresholds for early risk stratification of sepsis patients: <200, very low risk; >300, moderate risk; >500, high risk; and > 1000 ng/L, very high risk.

Compared with sepsis, there are only a few studies that have evaluated the clinical usefulness of presepsin in pneumonia.^[19]^ Klouche et al reported that presepsin was useful for differentiating severe community-acquired pneumonia from noninfectious respiratory failure.^[20]^ In other study, presepsin level on admission was a useful predictor of 30-day mortality and an additional prognostic biomarker on existing severity assessment scales in hospitalized patients with pneumonia.^[21]^

Recently, presepsin served as a useful prognostic biomarker for patients with COVID-19.^[11,22]^ In the present study, the mean admission presepsin levels were 1488 pg/mL in the MVHF-OT group, 1051 pg/mL in the C-OT group, and 654 pg/mL in the N-OT group (Table 2), thus well-correlated with disease severity.

The MDW reflects the size distribution of circulating monocytes.^[12]^ Unlike other sepsis biomarkers such as the CRP and PCT levels, the MDW is automatically reported (along with the complete blood count and differential counts) and can detect sepsis early.^[10,23]^ Elliott et al^[10]^ reported that an MDW > 20.0 (measured in the emergency department) indicated sepsis. The MDW is also affected by viral infection, including SARS-CoV-2 infection.^[23,24]^

Circulating monocytes and lymphocytes play important roles in immune surveillance and the inflammatory response. These cells are among the first to respond to viral infection; activated cells undergo morphological changes.^[25–27]^ Although there was no significant difference in the monocyte count among-group, the MDW at admission differed significantly by COVID-19 severity. One week later, there was no significant difference. Therefore, the MDW is a potential early predictor of COVID-19 severity and it can be easily checked with CBC.

Other studies found that decreased lymphocyte and platelet counts and elevated levels of CRP, PCT, D-dimer, LDH, liver enzymes, and creatinine were associated with poor outcomes in COVID-19 patients.^[8,9,28]^ However, in an Italian study featuring adjusted analysis, CRP was the only biomarker associated with increased risks of death and ICU admission.^[29]^ We found that decreased lymphocyte and platelet counts; an increased ESR; and increased CRP, PCT, LDH, and ferritin levels were associated with disease severity. The rises in inflammatory markers and acute phase reactants probably reflect the cytokine storm associated with severe infection and subsequent end-organ damage.^[30]^ In COVID-19 patients, an elevated PCT level, which serves as a biomarker of bacterial infections,^[31]^ suggests the possibility of such an infection.^[32]^

KL-6 is a high-molecular-weight mucin-like glycoprotein produced by type II pneumocytes and bronchial epithelial cells.^[33]^ KL-6 serves as a sensitive marker of interstitial lung diseases (ILDs) such as pulmonary fibrosis, connective tissue disease-associated ILD, hypersensitivity pneumonitis, and pulmonary sarcoidosis.^[34]^ Increased levels of KL-6 reflect greater lung damage and regeneration of type II pneumocytes. In other studies, severely ill COVID-19 patients evidenced higher serum KL-6 levels than did mild cases.^[35,36]^ Frix et al^[33]^ reported that the serum KL-6 levels in COVID-19 patients were higher than in healthy subjects but not as high as in ILD patients. However, we found no among-group differences in KL-6 levels at either admission or discharge. Additional studies are needed to adjust for confounding factors.

Our work has certain limitations. First, we retrospectively analyzed 87 COVID-19 inpatients treated at a single center. We included patients ranging from asymptomatic to severely ill (requiring extracorporeal membrane oxygenation) and patients were classified by clinical disease severity. Thus, the group numbers differed; the number of patients with severe disease was relatively small. Second, the Korean hospital admission criteria for COVID-19 patients changed during the pandemic. In the early days, all patients, regardless of disease severity, were admitted. However, as the number of patients increased, mildly ill patients were accommodated at residential treatment centers rather than hospitals. Therefore, hospital admission times and disease severity varied over time. Third, we lacked information on certain laboratory results. For example, presepsin was measured only at admission. We used only the admission values of PCT, LDH, and ferritin because some later values were lacking or were measured at different times after admission. Finally, several laboratory indicators were measured at admission irrespective of disease severity. However, detailed statistical validation was not conducted to determine whether the laboratory indicators of this study could be used as biomarkers in COVID-19 patients. Further studies are needed in the future. Nevertheless, this study presents valuable real-world data for characterizing a new pandemic infection that we have never experienced before.

In conclusion, we verified the utilities of known biomarkers, and we propose that the MDW and presepsin level may help clinicians to classify the severity of COVID-19, predict prognosis, and determine treatment.

## Acknowledgments

We greatly appreciate all the members of the Eunpyeong St. Mary’s Hospital for their efforts and devotion during the crisis of COVID-19.

## Author contributions

Conceptualization: all authors.

Data curation: Sei Won Kim, Heayon Lee and Sang Haak Lee.

Formal analysis: Sung Jin Jo, Jehoon Lee and Jihyang Lim.

Investigation: Sei Won Kim, Heayon Lee and Jihyang Lim.

Methodology: Sei Won Kim, Heayon Lee and Jihyang Lim.

Resources: Sei Won Kim, Heayon Lee and Sang Haak Lee.

Supervision: Sang Haak Lee Jehoon Lee and Jihyang Lim.

Validation: Sei Won Kim and Jihyang Lim.

Writing—original draft: Sei Won Kim.

Writing—review and editing: Heayon Lee and Jihyang Lim.
